# Bilateral exudative retinal detachment, unusual pattern of uveitis with multiple sclerosis

**DOI:** 10.1186/s12348-023-00337-2

**Published:** 2023-03-22

**Authors:** Rehab Sabry Helal, Saket Arya, Rami Abu Sbeit, Maha Elshafei

**Affiliations:** Hamad Medical Corporation, Ophthalmology department, Ambulatory Care Centre, Doha, Qatar

**Keywords:** MS associated uveitis, Exudative retinal detachment, Intermediate uveitis

## Abstract

MS (Multiple sclerosis) associated uveitis used to have limited phenotypes. Bilateral exudative retinal detachment has never been recognized as a pattern of MS-associated uveitis. We are reporting a patient with multiple sclerosis who presented initially with the usual pattern of intermediate uveitis and later developed bilateral exudative retinal detachment.

## Introduction

The prevalence of uveitis in Multiple sclerosis (MS) varies considerably between studies ranging from 0.4% to 26.9% [1]. It has been described in both relapsing–remitting MS (RRMS) and primary progressive MS [2]. While panuveitis was the common pattern in some studies [3], intermediate uveitis was the most common phenotype associated with MS in most reports [4, 5].

The association between intermediate uveitis and multiple sclerosis has always been recognized, as both share the same genetic risk factors, (HLA-DR2 and its split antigen HLA-DR15) explaining their relation [6].

Morphologically, multiple sclerosis associated intermediate uveitis and parsplanitis can’t be distinguished based on ocular features alone [7].

Bilateral exudative retinal detachment is not an acknowledged feature of MS associated uveitis.

## Case report

**Fig. 1:**
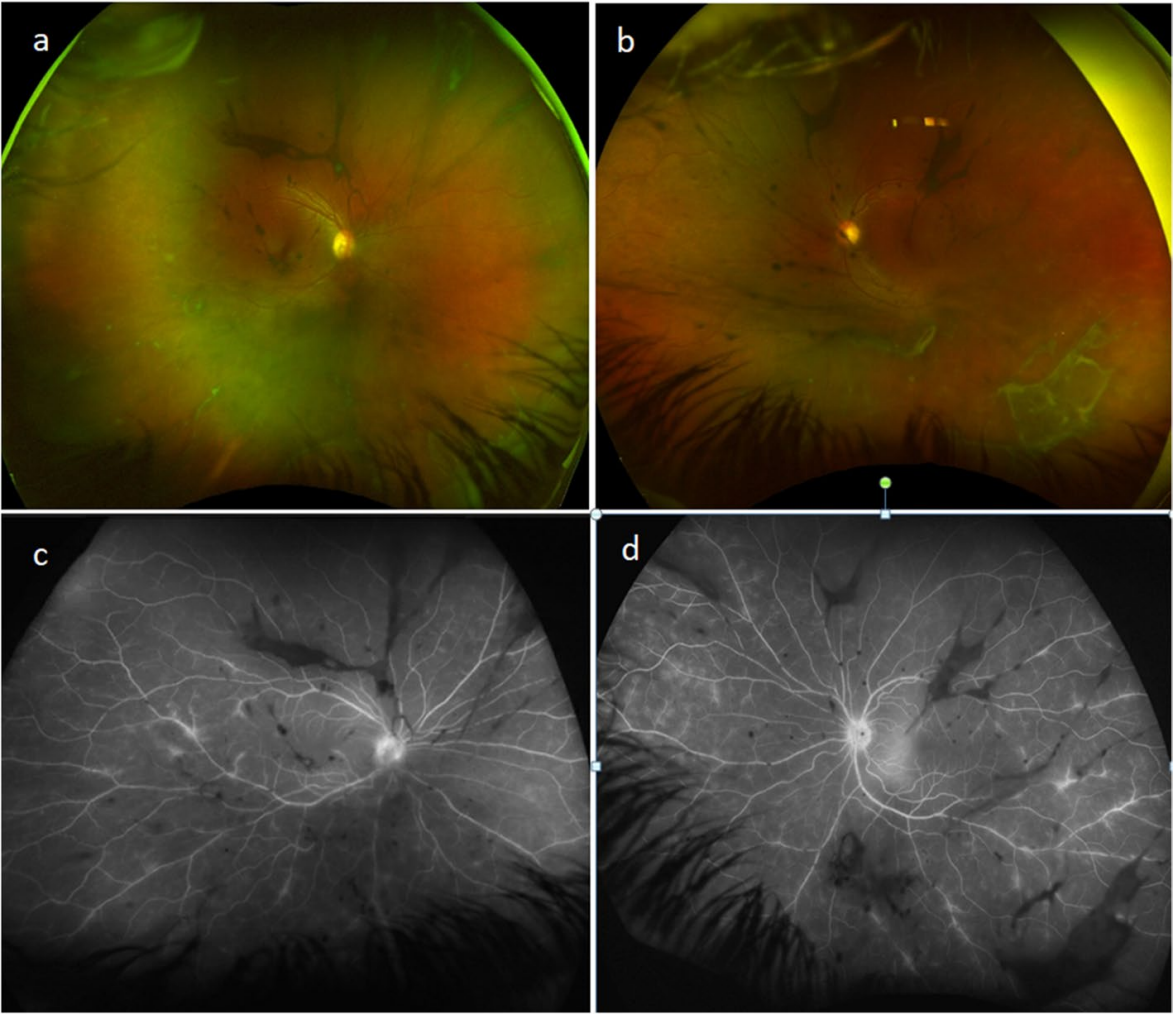
**a**, **b**: right and left eye wide field fundus photos showing coarse vitreous condensations and subtle peripheral vasculitis **c**, **d**: late venous phase wide field fundus fluorescein angiography showing bilateral diffuse peripheral capillaritis and non occlusive vasculitis involving secondary and tertiary veins with no retinal ischemia and no macular capillary leakage. Hyper fluorescence of the disc was noted in both eyes

A 30 years old Middle Eastern female, presented to our facility complaining of left eye floaters and blurring of vision for a few days. Her past history was free of any ocular or systemic illnesses. Her BCVA on presentation was 6/6 right eye and 6/9 left eye. The anterior segment showed variable size KPs at both corneas with + 1 and + 2 anterior chamber cells in both eyes. The posterior segments showed + 1 vitreous cells and significant vitreous condensations with mild peripheral vasculitis (Fig. 1a,b). She was diagnosed as a case of bilateral intermediate uveitis and advised to start topical steroids until her fundus fluorescein angiography and uveitis workup were done.

Blood workup did not reveal any positive results. ACE, lysozyme, syphilis serology and QuantiFERON TB test were all negative.

Magnetic Resonance Imaging (MRI) showed multiple cerebral white matter spots which were rounded and oval small patchy areas of T2/FLAIR bright signal. Dawson finger appearance was noticed in some lesions. Spinal cord showed intramedullary T2 bright lesion opposite T6-T7 vertebrae with no post contrast enhancement.

Cerebrospinal fluid (CSF) analysis showed oligoclonal bands and cell cytology showed small mature lymphocytes and Polymorphoneutrophils indicating mild cellular inflammation. NMO-IgG was negative.

Fundus fluorescein angiography (Fig. 1c,d) showed bilateral diffuse peripheral capillaritis and non occlusive vasculitis involving secondary and tertiary veins with no retinal ischemia or macular capillary leakage. Hyper fluorescence of the disc was noted in the late phase.

As the patient presented at the beginning of Covid-19 pandemic when there were no clear guidelines about the use and safty of systemic steroids and immunosuppressives and she did not show any obvious vision threatening pathology, she was observed with topical steroids only for 2 months then oral steroids were started at a dose of 60 mg after her floaters increased. She showed a good response to oral steroids and was advised to start azathioprine 50 mg as a steroid sparing agent (to be escalated later to 2.5 mg/kg/day), but her neurologist was planning to start interferon beta-1a and advised to stop azathioprine for fear of the synergistic myelosuppression effect of both medications, so azathioprine was stopped.

While she was tapering her oral steroids she presented with right eye sudden drop of vision and found to have rhegmatogenous retinal detachment reaching the macula with superotemporal retinal dialysis in spite of the total improvement of her intraocular inflammation. She underwent pars plana-vitrectomy and silicone oil tamponade then silicone oil was removed after 5 weeks.

She was followed by her neurologist with a diagnosis of RRMS (relapsing remitting multiple sclerosis) and was started on Interferon beta-1a (44mcg). Her follow up neuro-imaging showed new demyelinating lesions so interferon was stopped and she was shifted to Rituximab.

After 4 months she developed the same pattern of retinal dialysis in her left eye notwithstanding the previous peripheral laser barricade and clinically inactive intermediate uveitis. She underwent the same surgery for the left eye followed by cataract surgery both eyes for silicone oil induced cataract.

She was doing fine for 3 months after her surgeries which were secured with oral steroids. Her vision improved to 6/18 right eye and 6/9 in the left eye (as the detachment in the left eye was detected before involving the macula).

**Fig. 2:**
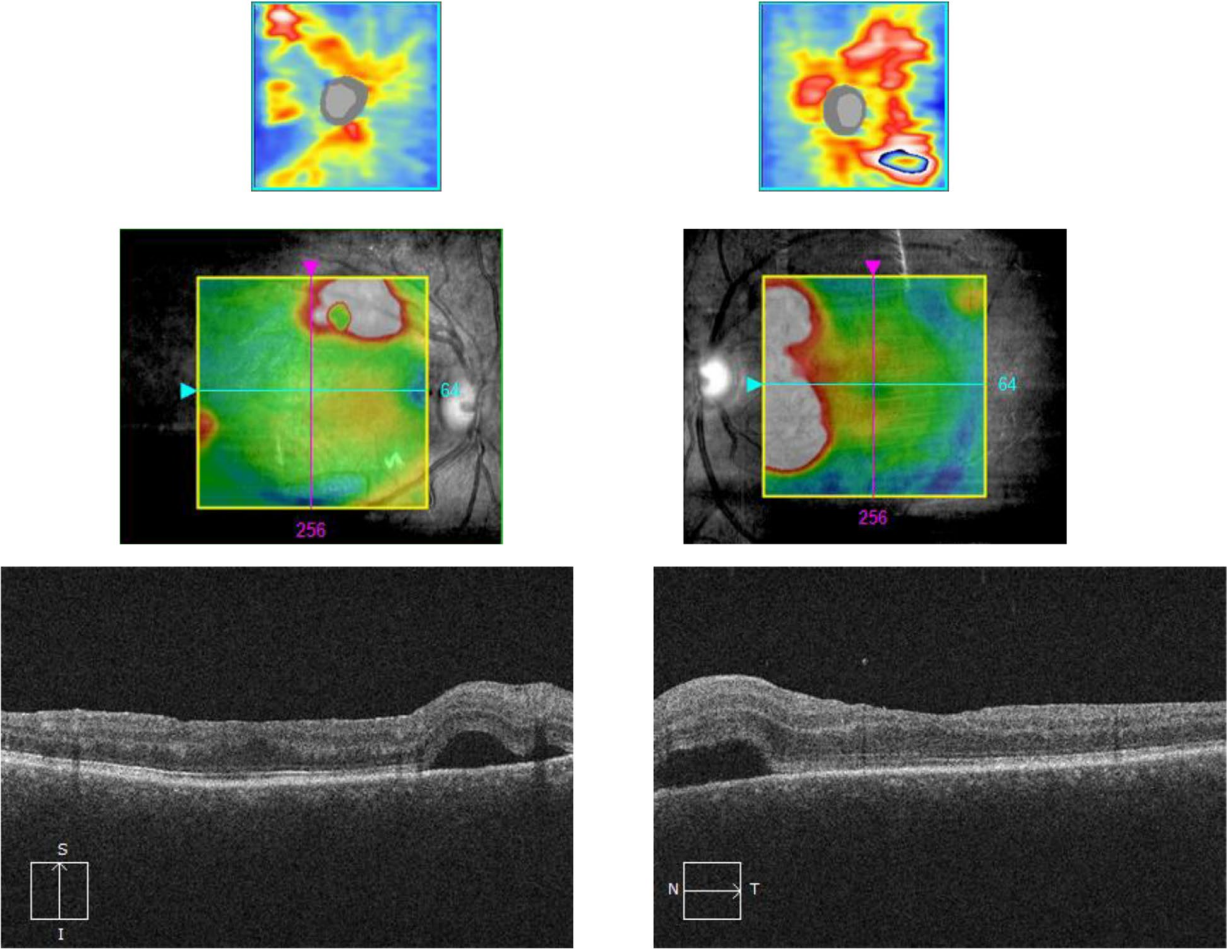
OCT (ocular coherence topography) of both eyes showing bilateral peripapillary multifocal subretinal fluid more in left eye

**Fig. 3:**
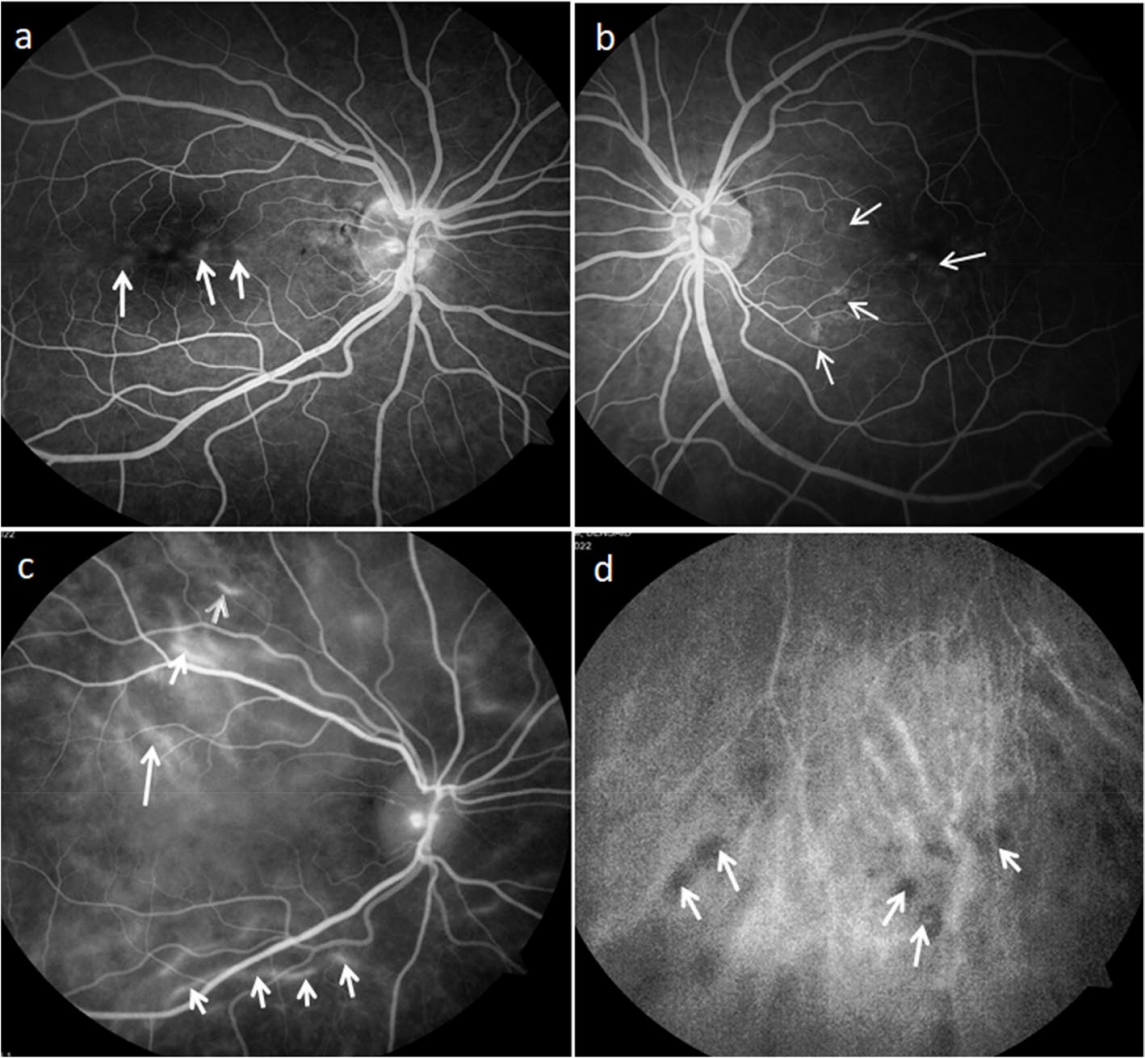
**a**, **b**: FFA (fundus fluorescein angiography) photos showing pinpoint leakage at the posterior pole of both eyes (arrows). **c**: ICG (indocyanine green angiography) of right eye showing hypercyanescence of the stromal choroidal vessels (arrows). **d**: ICG of the peripheral retina of left eye with multiple hypocyanescent dark dots (arrows) at the periphery

**Fig. 4:**
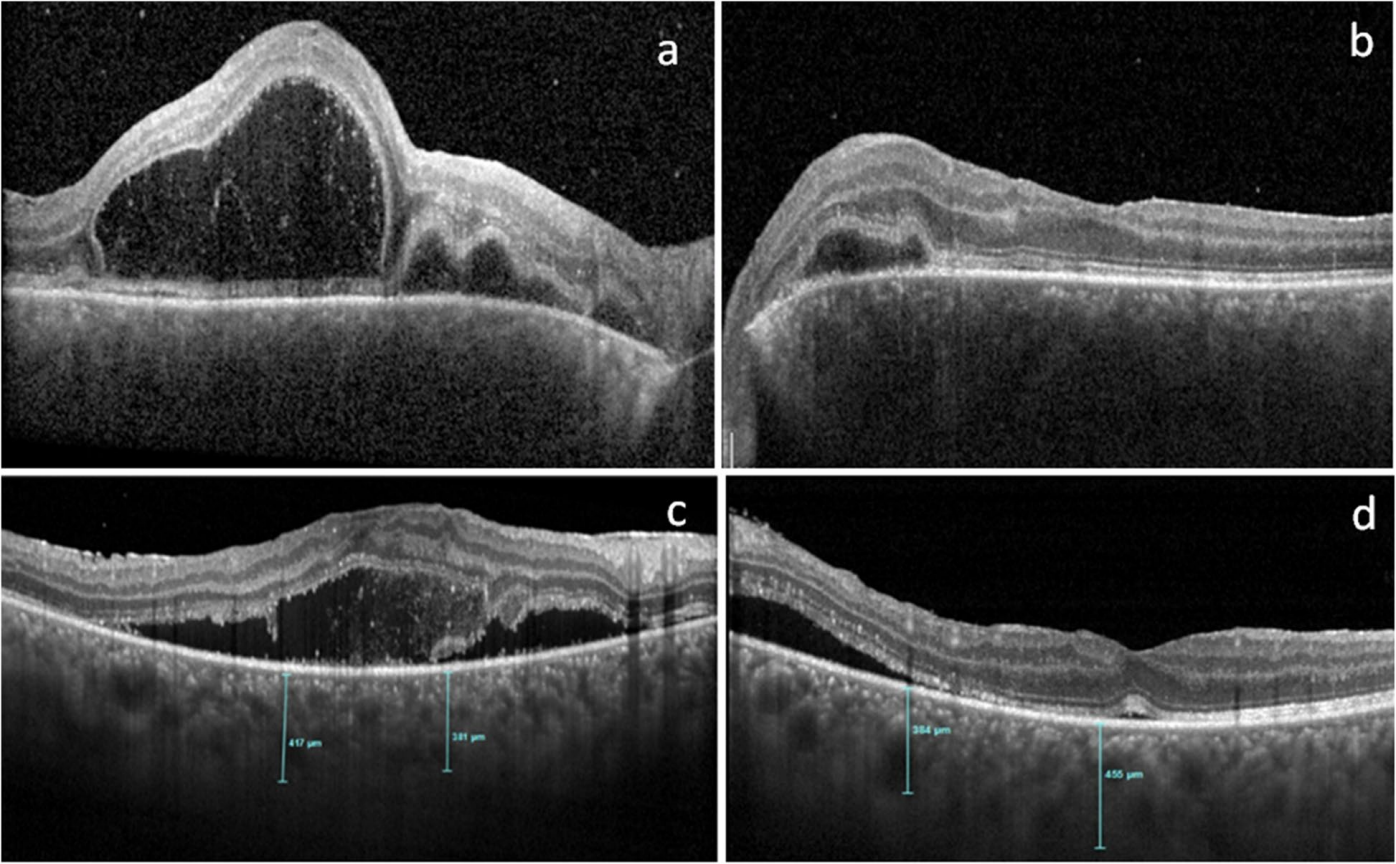
**a** and **b**: right and left eye enhanced depth imaging (EDI-OCT) showing beyond measurement choroidal thickening with exudative retinal detachment more in the right eye.**c** and **d**: EDI-OCT showing improvement in subretinal fluid and choroidal thickness (but still high, central choroidal thickness: 417 micron right eye and 456 micron left) after IV pulse therapy

During her regular follow up visit she was found to have bilateral mild peripapillary subretinal fluid (Fig. 2). FFA showed multiple pinpoint leakages at the posterior pole but no other pathology was noted (Fig. 3a, b). After 5 days she developed bilateral multifocal exudative retinal detachment with minimal cells in the anterior chamber and vitreous cavity. Flaremetry readings were 35 right eye and 44 left eye and choroidal thickness was beyond the measurement of Enhanced depth imaging (EDI) OCT (Fig. 4a and b). Upon questioning her, She did not report the usual prodromal signs of VKH, except for mild tinnitus which could be related to MS according to her neurologist. The patient was admitted for IV pulse steroid therapy and MRI was done and showed no deterioration of MS lesions and no evidence of meningeal or inner ear enhancement, which are usually reported with VKH. ICG was performed during her admission and revealed hypercyanecence of the stromal choroidal vessels (Fig. 3c) and multiple hypocyanescent dark dots at the periphery of both eyes (Fig. 3d).

She received 5 doses of IV methylprednisolone, 1 gm daily and then shifted to oral steroids and referred to rheumatologist to start immunosupressive therapy.

The exudative retinal detachment resolved completely with that treatment and her vision was stabilized on 6/18 right eye and 6/9 left eye. Currently she is on Rituximab injection and azathioprine (125 mg daily) and doing fine with no signs of active intraocular inflammation or MS.

## Discussion

Multiple sclerosis used to represent mainly as intermediate pattern of uveitis with peripheral retinal vasculitis. Anterior uveitis was also reported but it is not clear if it is a distinct entity or it can be a part of missed or less significant intermediate uveitis. Panuveitis with retinal vasculitis is a well known phenotype which can line up with the previous patterns. A case of CRAO (central retinal artery occlusion) which may represent the extreme of retinal vasculitis was reported with MS [8]. 

Other sporadic cases of unusual patterns were reported like a case of clinically definite multiple evanescent white dot syndrome (MEWDS) in multiple sclerosis patient [9]. 

Our patient presented with the classic granulomatous intermediate uveitis with peripheral retinal vasculitis and MS was on the top of the differential diagnosis so MRI was done early after infectious causes were excluded and her management was guided with the severity of her signs and symptoms which were not severe or vision threatening at the beginning, but latter on she came up with unexpected bilateral retinal dialysis then bilateral exudative retinal detachment.

Montero JA et al. [10] reported a case of 34 years old woman who was diagnosed with MS on the basis of bilateral lower limb weakness, ataxia, and optic neuritis, as well as compatible MRI findings and 2 years later she developed progressive bilateral visual loss associated with peripapillary exudative retinal detachment and other features of Vogt-Koyanagi-Harada (VKH) syndrome. She had decreased hearing in her left ear, neck stiffness and headache during the same period.

Our patient was different in some aspects; first, she presented initially with the classic pattern of intermediate uveitis and peripheral retinal vasculitis and then developed a completely different phenotype of uveitis with bilateral exudative retinal detachment involving the posterior pole. Second, she did not show the other features of VKH syndrome except for mild tinnitus which was not clear if it is related to VKH or MS disease. And later she did not show the usual cicatricial or pigmentary changes which usually pursue VKH. Brain MRI was done within 3 days of her symptoms and it did not show the usual VKH findings of meningeal or inner ear enhancement or white matter changes reported with these patients.

The surgical procedures of both eyes may also offer the differential diagnosis of sympathetic ophthalmia which was difficult to exclude but the absence of significant granulomatous anterior uveitis, vitritis and Dellen Fuchs nodules did not support the diagnosis of sympathetic ophthalmia. After 18 months of follow up; She did not develop the sunset glow fundus, focal RPE loss or subretinal fibrosis that are usually seen in sympathetic ophthalmia and VKH.

Sarcoidosis can cause both intermediate uveitis and choroidal involvement but her work up did not show any supportive radiological or laboratory findings and her ocular features were not pointing towards sarcoidosis with the absence of the usual peripheral chorioretinal granulomas which are usually seen in sarcoid intermediate uveitis.

Even the hypocyanescent dark dots which were recorded on the ICG did not leave on their wake any chorio-retinal scars which are usually anticipated with sarcoidosis.

VKH-like disease was previously reported with interferon alpha treatment [11] but it was not the scenario with our patient who was treated with interferon beta 1-a for 3 months only and it was stopped completely 7 months before the development of the exudative retinal detachment. Severe intermediate uveitis can be complicated with exudative retinal detachment but It starts at the inferior periphery extending from the inflamed pars plana but never at the posterior pole with a multifocal pattern. The severe choroidal thickening which was recorded in our patient cannot be explained in view of the pathogenesis of intermediate uveitis. The choroid was never been reported to be involved with MS-associated uveitis. We believe that the occurrence of the exudative retinal detachment after the resolution of the intermediate uveitis was a separate and different pathology but we are not sure if there is a direct correlation between the exudative retinal detachment and MS.

## Conclusion

Our patient could represent a new pattern of uveitis which was never reported with multiple sclerosis, or a probable VKH disease supporting the previous reports and suggestions of the link between MS and VKH. Further observation of such rare co-occurrence is required for clear understanding of the nature of MS associated uveitis.

## Data Availability

The data used in that case report is available from the corresponding author on reasonable request.
